# Health and Liver Diagnostic Markers Influencing Glycemia in Subjects with Prediabetes: Preview Study

**DOI:** 10.3390/diagnostics14242895

**Published:** 2024-12-23

**Authors:** Omar Ramos-Lopez, Diego Martinez-Urbistondo, Santiago Navas-Carretero, Ruixin Zhu, Maija Huttunen-Lenz, Gareth Stratton, Teodora Handjieva-Darlenska, Svetoslav Handjiev, Jouko Ensio Sundvall, Marta P. Silvestre, Elli Jalo, Kirsi H. Pietiläinen, Tanja C. Adam, Margriet Westerterp-Plantenga, Elizabeth Simpson, Ian MacDonald, Moira A. Taylor, Sally D. Poppitt, Wolfgang Schlicht, Jennie Brand-Miller, Mikael Fogelholm, Anne Raben, J. Alfredo Martinez

**Affiliations:** 1 Medicine and Psychology School, Autonomous University of Baja California, Tijuana 22390, Baja California, Mexico; oscar.omar.ramos.lopez@uabc.edu.mx; 2 Internal Medicine Department, Clinica Universidad de Navarra, 28027 Madrid, Spain; dmurbistondo@gmail.com; 3 Centre for Nutrition Research, Department of Nutrition, Food Science, Physiology and Toxicology, University of Navarra, 31009 Pamplona, Spain; jalfredo.martinez@imdea.org; 4 Spanish Biomedical Research Centre in Physiopathology of Obesity and Nutrition (CIBERobn), 28029 Madrid, Spain; 5 IdiSNA, Navarra Institute for Health Research, 31009 Pamplona, Spain; 6 Department of Nutrition, Exercise and Sports, Faculty of Science, University of Copenhagen, DK-2200 Copenhagen, Denmark; ruixinzhu@nexs.ku.dk (R.Z.); ara@nexs.ku.dk (A.R.); 7 Institute for Nursing Science, University of Education Schwäbisch Gmünd, 73525 Schwäbisch Gmünd, Germany; maija.huttunen-lenz@ph-gmuend.de; 8 Applied Sports, Technology, Exercise and Medicine (A-STEM) Research Centre, Swansea University, Swansea SA1 8EN, UK; g.stratton@swansea.ac.uk; 9 Department of Pharmacology and Toxicology, Medical University of Sofia, 1000 Sofia, Bulgaria; teodorah@abv.bg (T.H.-D.); svhandjiev@gmail.com (S.H.); 10 Finnish Institute for Health and Welfare, 00271 Helsinki, Finland; jouko.sundvall@thl.fi; 11 Human Nutrition Unit, Department of Medicine, School of Biological Sciences, University of Auckland, Auckland 1024, New Zealand; marta.silvestre@nms.unl.pt (M.P.S.); s.poppitt@auckland.ac.nz (S.D.P.); 12 CINTESIS, NOVA Medical School (NMS), Universidade Nova de Lisboa, 1169-056 Lisboa, Portugal; 13 Department of Food and Nutrition, University of Helsinki, 00014 Helsinki, Finland; elli.jalo@helsinki.fi (E.J.); mikael.fogelholm@helsinki.fi (M.F.); 14 Obesity Research Unit, Research Program for Clinical and Molecular Metabolism, Faculty of Medicine, University of Helsinki, 00014 Helsinki, Finland; kirsi.pietilainen@helsinki.fi; 15 Department of Nutrition and Movement Sciences, NUTRIM, School of Nutrition and Translational Research in Metabolism, Maastricht University, 6200 Maastricht, The Netherlands; t.adam@maastrichtuniversity.nl (T.C.A.); m.westerterp@maastrichtuniversity.nl (M.W.-P.); 16 MRC/ARUK Centre for Musculoskeletal Ageing Research, ARUK Centre for Sport, Exercise and Osteoarthritis, National Institute for Health Research (NIHR) Nottingham Biomedical Research Centre, Nottingham DE22 3DT, UK; liz.simpson@nottingham.ac.uk (E.S.); ian.macdonald1@nottingham.ac.uk (I.M.); 17 Division of Physiology, Pharmacology and Neuroscience, School of Life Sciences, Queen’s Medical Centre, Nottingham NG7 2UH, UK; 18 NIHR Nottingham Biomedical Research Centre at Nottingham University Hospitals NHS Trust and University of Nottingham, The David Greenfield Human Physiology Unit, Division of Physiology, Pharmacology and Neuroscience, School of Life Sciences, Faculty of Medicine and Health Sciences, University of Nottingham, Nottingham NG1 5DU, UK; moira.taylor@nottingham.ac.uk; 19 Exercise and Health Sciences, University of Stuttgart, 70569 Stuttgart, Germany; wolfgang.schlicht@f10.uni-stuttgart.de; 20 School of Life and Environmental Sciences and Charles Perkins Centre, University of Sydney, Sydney, NSW 2006, Australia; jennie.brandmiller@sydney.edu.au; 21 Precision Nutrition and Cardiometabolic Health, IMDEA Food Institute, CEI UAM+CSIC, 28049 Madrid, Spain; 22 Medicine and Endocrinology Department, Universidad de Valladolid, 47002 Valladolid, Spain

**Keywords:** prediabetes, adiposity, fatty liver, quality of life

## Abstract

Introduction: Glucose homeostasis may be dependent on liver conditions and influence health-related markers and quality of life (QoL) objective measurements. This study aimed to analyze the interactions of glycemia with liver and health status in a prediabetic population. Subjects and methods: This study included 2220 overweight/obese prediabetics from the multinational PREVIEW project. Anthropometrics; clinical, metabolic and other health-related markers; and QoL variables were analyzed. Univariate and multilinear-adjusted regression models were run to explain the interrelationships and effect modification between glycemia, health-related QoL (applying SF-12) and metabolic/liver health (using the HSI, a putative marker of fatty liver). Results: Relevant age/sex interactions were found concerning the levels of insulin, HOMA-IR, C peptide and transaminases in this prediabetic population. Multivariate models identified age, sex, glucose, WC and QoL as important predictors of HSI variability (adj. R value = 0.1393, *p* < 0.001), whereas the QoL status was statistically related to age, sex, HOMA-IR and HSI (adj. R value = 0.1130, *p* < 0.001) in this glycemia-impaired group. Furthermore, the QoL values declined with increased HSI scores, where a significant interaction was found (*p* = 0.011) when the data were analyzed when comparing lower glycemia vs. higher glycemia in prediabetics. Indeed, an effect modification was featured depending on the glycemia levels concerning the QoL and HSI worsening. Conclusion: Glycemia associations with the QoL status and liver metabolism markers were evidenced, with clinical implications for diabetes and liver disease precision management given the modification of the QoL outcomes depending on the liver status and glycemia concentrations. Notably, independent associations of circulating glucose with age, sex, adiposity, inflammation and C peptide levels were found.

## 1. Introduction

Type 2 diabetes mellitus (T2DM) is a chronic metabolic disease featuring persistent elevated blood glucose levels (hyperglycemia) resulting from impaired insulin secretion or resistance to peripheral actions of insulin in target tissues [1]. In this context, prediabetes is identified as a state of intermediate hyperglycemia, but not meeting the diagnosis criteria for diabetes [2]. Hyperglycemia is a phenomenon observed in essentially all individuals with diabetes and is associated with a hormonal balance dysregulation (including insulin, glucagon and cortisol impairments), increased hepatic glucose output and diverse metabolism dysfunctions, affecting multiple organs and body systems [3].

Because prediabetes is a highly heterogeneous metabolic state, phenotype stratification and mediation factor analyses are important for precision medicine to improve risk prediction, prognosis and disease management [4]. Thus, prediabetes characterization includes different biomarkers, such as impaired fasting glucose (IFG), impaired glucose tolerance (IGT) and elevated glycated hemoglobin (HbA1c), under criteria formulated by several international committees [5], where other pathways may also be disturbed.

Indeed, quality of life (QoL), a subjective evaluation to capture global health, well-being and unspecific morbidities, has been explored as a potentially diagnostic tool in many chronic metabolic diseases including T2DM [6]. Indeed, QoL research has increased in recent years since it has been recognized as a health marker and an important endpoint in medical domains [7]. As in other chronic diseases, evaluating QoL is critically important since wellbeing is the ultimate goal of diabetes care, improving self-care management, adherence to prescribed medication lifestyle modification and interactions with other health markers [8].

Furthermore, the liver has a recognized role in the control of carbohydrate/lipid metabolism and other diverse metabolic functions [9]. In the presence of hepatic disease, the homeostasis of glucose and fatty acids is impaired as a consequence of disorders, including insulin resistance and gluconeogenesis, which are associated with hyperglycemia, compensatory hyperinsulinemia and hypertriglyceridemia [10]. In fact, the new consensus about steatosis liver disease [11] reinforces the importance to study the liver as a central axis in the development of metabolic diseases, whose understanding concerning physiopathological relationships with glycemia and health status based on QoL assessment will pave the way for precision medicine. Indeed, the feasibility of looking for new markers in the diagnosis of prediabetes in order to prevent liver damage has led to the proposal of potential new markers [12].

The aim of this research was to analyze the relationship between glycemia on the liver status and health-related markers, including QoL in a population with prediabetes, to understand the factors involved in such interrelationships and also the conjoint interpretation of independent glycemia determinants in prediabetics.

## 2. Materials and Methods

### 2.1. Population

The present ancillary study was based on the PREVIEW project (https://preview.ning.com/, accessed on 23 February 2024), a large multi-center international trial addressing the prevention of T2DM through lifestyle (diet and exercise) intervention in adults with overweight (BMI ≥ 25–29.9 kg/m^2^) or obesity (BMI ≥ 30–40 kg/m^2^), and suffering from prediabetes. The design, methods and ethical issues of the PREVIEW study, as well as the selection criteria for the enrolled adults, have been described in detail elsewhere [13]. Basically, the participants had to present with prediabetes, understood as high baseline glucose levels (5.6–6.9 mmol/L), impaired glucose tolerance (7.8–11.0 mmol/L at 2 h after oral administration of 75 g glucose) or HbA1c (5.7 to 6.4%), in addition to suffering from overweight or obesity. The PREVIEW Study is registered at clinicaltrials.gov (identifier code: NCT01777893).

Following the intervention design, after 8 weeks of weight loss, the main goal of the PREVIEW Study was to maintain the weight lost during the next 34 months, following a 2-factor (diet × physical activity) lifestyle intervention consisting of a diet high in protein or moderate in protein, and with moderate or intensive physical activity [13]. To achieve the final number of 2326 participants with prediabetes and presenting overweight or obesity, a total of 15,611 subjects were pre-screened, 5472 screened, and 2326 finally randomized and enrolled in this study. Of those randomized, 2223 subjects attended the baseline measures and 962 men and women finished the 3-year intervention. All centers had approval from their respective Research Ethics Boards before starting the trial [13], and all participants were required to give written informed consent in their mother tongue before enrollment. In this investigation, quality of life [14] and liver status [15] as global health surrogates, were specifically measured.

### 2.2. Study Variables and Data Management

Baseline variables of the adult population were reasonably requested to the PREVIEW project database administrator in Denmark. All data are stored in a central project database at the University of Copenhagen. The central database ensured standardized handling and storing of data, as well as the delivery of data both within and after the official project period (2013–2018).

The database received input from four data sources on a regular basis: (1) All immediate data measured (e.g., anthropometrics, blood glucose) and interviewed (e.g., use of medication) during the CIDs and entered into the OpenClinica server (electronic case report form). (2) Data on social-cognitive determinants of behavior, cultural and socio-demographics, and socio-economic components were collected by the questionnaire delivery platform (QDP) designed for PREVIEW by NetUnion. The participants entered their own data into the QDP. A paper version of the questionnaires was also available. (3) Physical activity was reported using the Baecke inventory, and an electronic physical activity log (PAL) was designed by Swansea University and the University of Stuttgart and implemented by NetUnion. (4) The Central Lab at the National Institute for Health and Welfare (THL) entered all the laboratory analyses into the data hub [13].

The data used within this ancillary study included anthropometrical, clinical and metabolic markers. In this context, weight was measured through calibrated scales to the nearest 0.1 kg, while height was measured with a stadiometer to the nearest 0.1 cm, and then the body mass index was calculated. Similarly, waist circumference was measured to the nearest 0.1 cm using an inelastic tap. For clinical markers, a digital sphygmomanometer was used to measure the resting systolic and diastolic blood pressures [13]. Metabolism markers included lipid, glucose, insulin and inflammatory determinations, which were collected and analyzed following standardized protocols specifically designed for this study [13].

The Homeostatic Model Assessment for Insulin Resistance (HOMA-IR) index was calculated as fasting insulin (μU/L) × fasting glucose (nmol/L)/22.5 [16]. The triglyceride-glucose index (TyG index) was computed as ln[TG (mg/dL) × glucose (mg/dL)/2], as described elsewhere [17]. The hepatic steatosis index (HSI) was estimated according to the following formula: 8 × (ALT/AST) + BMI + 2 (if T2D) + 2 (if female), as previously reported [15]. A cutoff value of HSI > 36 was established for the screening of fatty liver, as defined elsewhere [18]. QoL information was collected using the SF-12 survey, as validated for a comparable population [19]. This survey offers a vision on the subjects’ self-perceived QoL, covering both the mental and physical score [19].

### 2.3. Statistical Analyses

Due to the large number of participants, continuous variables are presented as means ± standard deviations. Parametric tests were applied since the main variables (glycemia, lipid, insulin and liver metabolism markers) were normally distributed (*p* > 0.05). Moreover, 2 × 2 factorial ANOVA tests were accordingly used to compare the anthropometrical, clinical and biochemical variables by age (median) and sex. Univariate linear regression models were run to assess the age/sex statistical interactions regarding metabolic status, as well as explore putative interrelationships between fasting glucose, liver and several health estimators, including QoL. Bonferroni-adjusted multiple linear regression models were also computed for predicting the QoL and HSI variability, as well as the contributions of several health-related markers. Regional differences were introduced as a potential confounding variable but were discarded once the result was non-significant. All data were analyzed using the IBM SPSS software, version 20 (IBM Inc, Armonk, NY, USA), and STATA/SE software, version 12.0, 2011 (StataCorp LLC, College Station, TX, USA www.stata.com). A *p*-value lower than 0.05 was considered statistically significant in all analyses. The differential statistical approach for seeking the interactions between variables was conceptually oriented as described elsewhere [20].

## 3. Results

Clinical and metabolic characteristics of the total population categorized by age (median) and sex groups are shown, and statistical comparison and age/sex interactions are reported (Table 1). Additional analyses that separated the population by age decades are reported in Supplementary Table S1. These additional analyses did not vary in a relevant way from the median analysis performed (Table 1).

The men younger than 55 years had higher central adiposity, glucose, insulin resistance, transaminases and blood pressure than the women, who, in turn, presented with more hepatic steatosis (measured by HSI) and a higher ALT/AST ratio (Table 1). Similar results were found within the group older than 55 years, but the women had a lower QoL than the men (Table 1). Interestingly, significant age and sex statistical interactions (effect modifications) were found for the values of insulin, HOMA-IR, C-peptide (C-Pep), aspartate aminotransferase (AST), alanine transaminase (ALT) and systolic blood pressure (SBP), with relevant roles in glucose homeostasis (Table 1). Regarding glycated hemoglobin (HbA1c), despite the differences regarding sex, the most significant were related to age (Table 1), although they were not too clinically relevant.

Simple and multiple linear regression models predicting the HSI and QoL are shown (Table 2 and Table 3, respectively).

Regarding the simple linear regression (Table 2), age, sex, glucose, waist circumference (WC) and QoL were significant predictors of HSI variability (Table 2, model 5).

Regarding QoL (Table 3), apparently the QoL status was explained by age, sex, HOMA-IR and HSI (Table 3, model 3).

**Figure 1:**
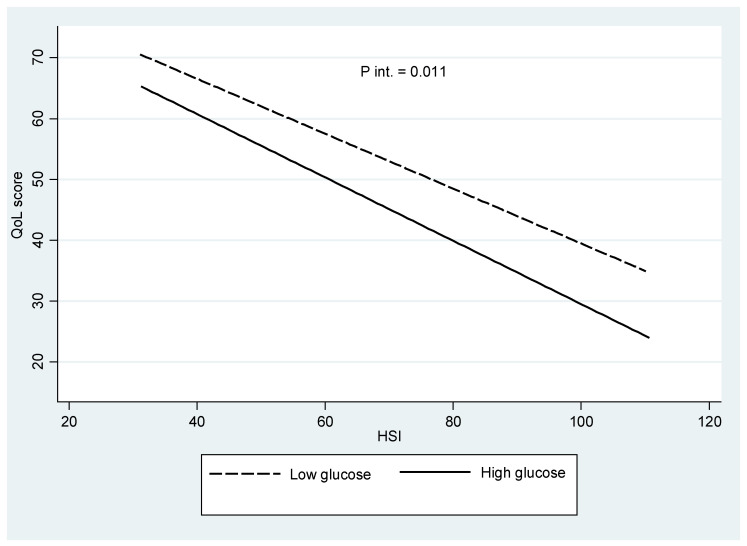
Interrelationships between QoL, HSI and blood glucose in individuals with prediabetes.

The interrelationships between QoL, HSI and glycemia are illustrated (Figure 1). Of note, the QoL values declined with increased HSI scores, where a significant interaction was found (*p* = 0.011) when the data were analyzed by comparing lower glycemia vs. higher glycemia (based on median values) in individuals with prediabetes. Indeed, an effect modification was shown that depended on the glycemia levels concerning differential QoL and HSI worsening.

**Figure 2:**
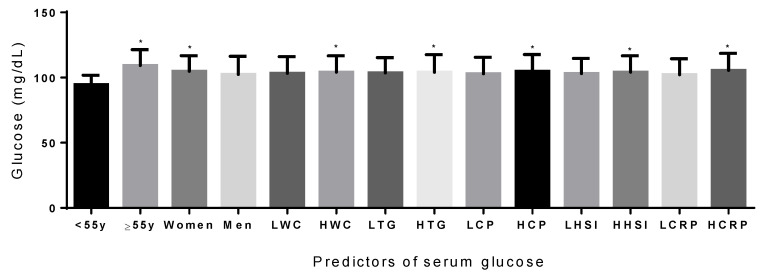
Independent relevant predictors of serum glucose. *p*-values were adjusted for multiple comparison using the Bonferroni test. LWC: low waist circumference; HWC: high waist circumference; LTG: low triglycerides; HTG: high triglycerides; LCP: low C-peptide; HCP: high C-peptide; LHSI: low hepatic steatosis index; HHSI: high hepatic steatosis index; LCRP: low C-reactive protein; HCRP: high C-reactive protein. * *p* < 0.05.

Furthermore, concerning the examination of the predictors of circulating glucose, the following parameters were found to anticipate higher levels: being older than 55 years; women; and individuals with higher WC, triglycerides, C-Pep, HSI and hs-CRP as independent contributors (Figure 2).

## 4. Discussion

Maintaining blood glucose homeostasis is important for optimal organ functioning, as well as analyzing the factors and interactions involved in glycemia regulation for health outcomes [21], which has been scarcely researched so far in prediabetes conditions [22]. Indeed, developing diagnostic markers related to prognostic outcomes are important for detecting metabolic pathways [12,23].

The role of QoL as a global indicator of health, as well as interactions with liver status concerning glucose utilization [15], has apparently never been addressed in subjects with prediabetes. The main finding of this study was the interaction (effect modification) found concerning the QoL and HSI depending on the glycemia levels, where the QoL declined more in individuals with higher glycemia and HSI scores compared with lower glycemia values, which suggests relevant interrelationships between these factors, with clinical implications for diabetes and liver disease management.

Age and sex are important variables in personalized precision medicine since they may contribute to optimize the effects of administered therapies and reduce adverse outcomes in many metabolic diseases, including diabetes and liver disease [24]. In this population of patients with prediabetes, relevant age and sex interactions concerning insulin, HOMA-IR, C-Pep and liver metabolic markers were found; these were also found when analyzed by decades of age, not only by the median, which are relevant confounding/mediating factors that should be considered in the clinical interpretation and the prescription of particular recommendations in prediabetic groups. In this context, previous reports from the PREVIEW trial revealed that the effects of a long-term lifestyle intervention on body weight and cardiometabolic health markers were dependent on age and sex in prediabetic adults [25]. Furthermore, adverse differences in cardiometabolic risk factors between subjects with prediabetes and normal glucose metabolism were found to be more pronounced in women than in men, as reported within the Maastricht Study [26].

The QoL score is a validated instrument to subjectively evaluate the perception about the influence of health and personal features on human well-being, which may be used as an integrative marker of health status [7]. In this investigation, QoL was sensitive to HSI and glycemia in this investigation, finding lower QoL scores in conditions of hyperglycemia and liver steatosis. In line with our results, prediabetes and T2DM were negatively associated with measures of QoL, which were similar between women and men [27]. Also, age, diabetes duration and fasting blood glucose level were inversely associated with QoL in adults [28]. Similarly, people with insulin resistance have worse QoL profiles in terms of social and physical functioning, emotional role limitations, pain and general health perception [29]. In this context, while the SF-12 QoL measure is validated cross-culturally, potential variations in self-reported QoL may arise due to cultural differences between the eight participating countries in the PREVIEW study.

Furthermore, it has been reported that patients with metabolic dysfunction-associated steatotic liver disease (MASLD) presented significantly poorer QoL in comparison with healthy controls, especially regarding the physical component of health [30]. Remarkably, it was postulated that the assessment of liver status by the HSI might have an impact on the health-related QoL response to lifestyle changes in subjects with metabolic syndrome [31], but never reported in subjects with prediabetes.

The HSI has been accepted as a simple, efficient and noninvasive tool to assess hepatic steatosis, being correlated with insulin resistance [15]. In this study, the HSI was predicted by age, sex, glucose, WC and QoL score, suggesting that liver steatosis, which is related to glucose/lipid metabolism, adiposity and insulin resistance, could be an early predictor of future metabolic complications. Consistently, a significant correlation between hepatic steatosis (as measured by the HSI) and dyslipidemia, overweight, obesity and hypertriglyceridemia has been reported in diabetic patients [32]. The HSI as a biomarker-based algorithm developed as a proxy for MASLD was also associated with cardiometabolic risk factors in a general Caucasian population [33]. Of note, estimated fatty liver disease using the HSI criteria, in conjunction with metabolic syndrome features and prediabetes, demonstrated a valuable clinical role to discriminate the patient risk of T2DM through the description of independent glucose metabolic remodeling phenotypes [34]. Additionally, hepatic steatosis scores (including HSIs) were used to estimate the response rate to lifestyle intervention in adults with obesity and/or diabetes [35]. Given the increased risk of adverse outcomes, the importance of regular prediabetes and diabetes screening in MASLD and the adoption of early lifestyle modifications to reduce disease progression should be highlighted [36]. Moreover, an interactive role of surrogate liver fibrosis with insulin resistance on the incidence of major cardiovascular events has been reported [36]. In addition to these issues, the psychological aspects affecting the adherence to a therapeutic approach must also be considered [37]. Regarding this point, the influence of psychological status, together with QoL, may positively or negatively influence the adherence to a prescribed treatment [38]. Indeed, a very important finding of this research was that glycemia was affected by age, sex, adiposity markers, inflammation, liver and insulin markers in people with prediabetes in an independent manner to that evidenced in diabetes [39].

On one hand, the strengths of this research included a high number of subjects with prediabetes analyzed with validated criteria that belonged to the multicenter PREVIEW study and facilitated investigating an intermittent/unstable stage before diabetes. Moreover, potential statistical interactions by age and sex were screened, as well as multiple comparison adjustments (under Bonferroni’s corrections) in the main outcome analysis. On the other hand, some limitations of this study encompassed the scarcity regarding exhaustive pharmacological therapies for metabolic alterations (i.e., dyslipidemia and blood pressure), as well as information concerning accurate alcohol consumption, smoking habit, and physical exercise as potential confounding factors in the predictive models.

The recognized relationship between liver health and T2D suggest that screening with clinically available scores may be an important approach to reducing disease costs [40]. Thus, interventions to reduce liver fat have the potential to improve insulin regulation in prediabetes [41], while the normalization of glycemia has been associated with health benefits and reduced incidence in related comorbidities [42]. Therefore, deep phenotyping and additional pathophysiology information is giving support toward precision medicine [39]. As an example, prediabetes defined by FPG and/or HbA1c showed differences according to sex concerning high-density lipoprotein cholesterol [43]. Moreover, changes in liver markers may anticipate variations in insulin resistance and glycemia, given that transaminases provide a pathophysiological core in forecasting diabetes occurrence [44].

As a summary, our research was focused on subjects with prediabetes (a really difficult population to recruit), which is a major difference between currently published research and available information. Furthermore, the independent associations of liver status and quality of life with glycemia were evidenced after adjustments by appropriate variables and confounding factors, which have seemingly never been reported with a comparable approach to our experimental design, while the interaction outcome is a pioneering principal finding since it has been shown for the first time that the factorial relationship of glycemia and liver status with quality of life involved three directions and permitted reaching the following conclusions: quality of life was negatively affected by increased HSI values, where quality of life was lower in more advanced hyperglycemic conditions and a paramount finding was the statistically significant modification of quality of life that was found when both glycemia and HSI values were more deteriorated with a more sharpened decline in the slope of the predicted quality of life dimensions in the group with higher (over the median) circulating glucose levels and advanced liver status damage.

Another relevant attraction of this contribution is that the results demonstrated the independent involvement of adiposity, lipid metabolism, insulin synthesis, and inflammation measurements and markers that modulated glycemia levels before diabetes emergence.

## 5. Conclusions

In conclusion, this research concerning liver and health markers in people with prediabetes have translational and multidisciplinary implications for diagnosis, prognosis and treatment approaches. Indeed, relevant interrelationships between glycemia homeostasis and associated markers, QoL and liver status were specifically evidenced in subjects with prediabetes, which were demonstrated that adiposity (WC), lipid metabolism (TG), insulin and inflammation (C-reactive protein) together and independently are indicators of circulating glucose in a population with prediabetes. This fact led the authors to consider this translational research in applications for individualized precision management of glycemia dysfunctions. Moreover, the QoL was affected conjointly by the liver status and glycemia levels, including a modification of the effect depending on the glucose concentration, where the QoL declined more in individuals with higher glycemia with increased HSI deterioration than with lower glycemia values. Indeed, investigations relative to the liver health and quality of life of patients with prediabetes are of value for the diagnosis and predictors of their metabolic consequences, particularly when considering that higher liver steatosis assessed by the HSI was associated with a lower quality of life, as well as a higher glucose concentration, where a statistical interaction between these two factors was demonstrated.

## Figures and Tables

**Table 1:** Clinical characteristics of the total population evidencing interactions of age and sex with glucose homeostasis and hepatic markers in prediabetics.

		<55 Years			≥55 Years					
Variable	Total	Women	Men	*p*	Women	Men	*p*	*p* Sex	*p* Age	*p* Int.
N	2220	783	317	-	719	401	-	-	-	-
BMI (kg/m^2^)	36 ± 6	37 ± 6	36 ± 7	0.287	35 ± 6	34 ± 5	**0.017**	**0.004**	**<0.001**	0.657
WC (cm)	110 ± 14	108 ± 15	117 ± 15	**<0.001**	107 ± 13	116 ± 13	**<0.001**	**<0.001**	0.151	0.318
Glucose (mg/dL)	104 ± 13	103 ± 12	106 ± 13	**0.012**	103 ± 13	104 ± 15	0.308	**0.016**	0.246	0.108
Insulin (μU/L)	13 ± 8	14 ± 8	16 ± 11	**<0.001**	12 ± 7	14 ± 7	**<0.001**	**<0.001**	**<0.001**	**0.027**
HOMA-IR	3.4 ± 2.1	3.5 ± 2.2	4.5 ± 3.2	**<0.001**	3.1 ± 1.8	3.5 ± 1.9	**0.002**	**<0.001**	**<0.001**	**0.002**
C-Pep (pmol/L)	913 ± 360	916 ± 373	956 ± 402	0.120	921 ± 352	860 ± 301	**0.004**	0.320	0.060	**0.002**
HbA1c (%)	5.4 ± 0.8	5.3 ± 1.1	5.4 ± 0.6	**0.003**	5.5 ± 0.5	5.4 ± 0.7	**<0.001**	0.357	**<0.001**	0.148
TyG index	8.6 ± 0.4	8.7 ± 0.5	8.8 ± 0.5	**0.026**	8.7 ± 0.5	8.8 ± 0.5	**0.002**	**<0.001**	0.601	0.076
TC (mg/dL)	200 ± 39	201 ± 40	203 ± 38	0.559	198 ± 37	202 ± 41	0.086	0.113	0.135	0.677
Triglycerides (mg/dL)	133 ± 69	134 ± 71	140 ± 71	0.202	127 ± 68	138 ± 70	**0.017**	**0.013**	0.403	0.376
HDL-c (mg/dL)	48 ± 10	49 ± 11	49 ± 10	0.587	49 ± 11	50 ± 10	0.724	0.959	0.567	0.650
LDL-c (mg/dL)	125 ± 32	125 ± 33	127 ± 32	0.504	124 ± 31	126 ± 34	0.328	0.276	0.287	0.792
hs-CRP (mg/L)	5.3 ± 3.0	5.4 ± 8.3	5.2 ± 5.2	0.645	5.4 ± 6.5	5.0 ± 6.4	0.331	0.309	0.169	0.803
AST (IU/L)	27 ± 10	25 ± 9	32 ± 12	**<0.001**	28 ± 10	30 ± 12	**0.010**	**<0.001**	0.201	**<0.001**
ALT (IU/L)	28 ± 15	24 ± 14	37 ± 17	**<0.001**	26 ± 14	29 ± 16	**0.002**	**<0.001**	**0.007**	**<0.001**
AST/ALT ratio	1.2 ± 0.6	1.3 ± 0.7	1.0 ± 0.61	**<0.001**	1.3 ± 0.7	1.2 ± 0.6	0.172	**<0.001**	0.054	0.051
SBP (mmHg)	129 ± 16	122 ± 14	131 ± 13	**<0.001**	132 ± 16	135 ± 16	**0.016**	**<0.001**	**<0.001**	**0.008**
DBP (mmHg)	78 ± 11	76 ± 11	80 ± 10	**<0.001**	78 ± 11	82 ± 10	**<0.001**	**<0.001**	**<0.001**	0.107
HSI	47 ± 8	50 ± 10	45 ± 9	**<0.001**	47 ± 8	44 ± 7	**<0.001**	**<0.001**	**<0.001**	0.243
QoL score (0–100)	60 ± 18	56 ± 19	57 ± 19	0.393	63 ± 19	67 ± 16	**<0.001**	**<0.001**	**<0.001**	0.690

Values are presented as means ± standard deviations. Bold numbers indicate *p* < 0.05. BMI: body mass index; WC: waist circumference; SBP: systolic blood pressure; DBP: diastolic blood pressure; HOMA-IR: homeostatic model assessment of insulin resistance; C-Pep: C-peptide; TC: total cholesterol; TG: triglyceride; HDL-c: high-density lipoprotein cholesterol; LDL: low-density lipoprotein cholesterol; hs-CRP: high-sensitivity C-reactive protein; AST: aspartate aminotransferase; ALT: alanine transaminase: TyG: triglyceride glucose index; HSI: hepatic steatosis index.

**Table 2:** Linear regression models predicting HSI variability in prediabetic subjects.

	B Coefficient (95% CI)		*p*
HSI (model 1)		Adjusted R value = 0.1026	**<0.001**
Age (years)	−0.1817 (−0.2269, −0.1364)		**<0.001**
Sex (women)	−5.2263 (−6.1386, −4.3141)		**<0.001**
Glucose (mg/dL)	0.0590 (0.0183, 0.0996)		**0.004**
HSI (model 2)		Adjusted R value = 0.0722	**<0.001**
Age (years)	−0.1013 (−0.1348, −0.0677)		**<0.001**
Sex (women)	−4.1384 (−4.9891, −3.2878)		**<0.001**
WC (cm)	0.0374 (0.0104, 0.0644)		**0.007**
HSI (model 3)		Adjusted R value = 0.1042	**<0.001**
Age (years)	−0.0651 (−0.0988, −0.0314)		**<0.001**
Sex (women)	−3.9255 (−4.7623, −3.0887)		**<0.001**
QoL score	−0.0902 (−0.1111, −0.0693)		**<0.001**
HSI (model 4)		Adjusted R value = 0.0925	**<0.001**
Age (years)	−0.0385 (−0.0727, −0.0043)		**0.017**
Sex (women)	−3.9823 (−4.8621, −3.1026)		**<0.001**
HOMA-IR	0.7842 (0.6085, 0.9599)		**<0.001**
HSI (model 5)		Adjusted R value = 0.1393	**<0.001**
Age (years)	−0.1410 (−0.1878, −0.0942)		**<0.001**
Sex (women)	−4.8923 (−5.8147, −3.9699)		**<0.001**
Glucose (mg/dL)	0.0522 (0.0110, 0.0933)		**0.013**
WC (cm)	0.0493 (0.0185, 0.0801)		**0.002**
QoL score	−0.0867 (−0.1106, −0.0629)		**<0.001**

Bold numbers indicate *p* < 0.05. WC: waist circumference; QoL: quality of life; C-Pep: C-peptide; HOMA-IR: homeostatic model assessment of insulin resistance; hs-CRP: high-sensitivity C-reactive protein; HSI: hepatic steatosis index.

**Table 3:** Linear regression models predicting QoL variability in prediabetic subjects.

	B Coefficient (95% CI)		*p*
QoL (model 1)		Adj. R value = 0.0766	**<0.001**
Age (years)	0.2678 (0.1942, 0.3413)		**<0.001**
Sex (women)	3.6912 (1.8207, 5.5617)		**<0.001**
HOMA-IR	−1.5522 (−1.9354, −1.1690)		**<0.001**
QoL (model 2)		Adj. R value = 0.0970	**<0.001**
Age (years)	0.3367 (0.2651, 0.4084)		**<0.001**
Sex (women)	1.1810 (−0.6735, 3.0356)		0.212
HSI	−0.4236 (−0.5219, −0.3254)		**<0.001**
QoL (model 3)		Adj. R value = 0.1130	**<0.001**
Age (years)	0.4249 (0.3265, 0.5233)		**<0.001**
Sex (women)	2.4300 (0.3959, 4.4640)		**0.007**
HOMA-IR	−1.3375 (−1.7570, −0.0180)		**<0.001**
HSI	−0.3940 (−0.5022, −0.2857)		**<0.001**

Bold numbers indicate *p* < 0.05. WC: waist circumference; QoL: quality of life; C-Pep: C-peptide; HOMA-IR: homeostatic model assessment of insulin resistance; hs-CRP: high-sensitivity C-reactive protein; HSI: hepatic steatosis index.

## Data Availability

Data used for this project are available from the corresponding author upon reasonable request.

## Supplementary Materials

The following supporting information can be downloaded at: https://www.mdpi.com/article/10.3390/diagnostics14242895/s1. Table S1: Clinical characteristics of the total population demonstrating interactions of age and sex with glucose homeostasis and hepatic markers in prediabetics.
