# Supplementary material for: Health and Liver Diagnostic Markers Influencing Glycemia in Subjects with Prediabetes: Preview Study
Source: Diagnostics (Basel). 2024 Dec 23;14(24):2895. doi: 10.3390/diagnostics14242895 (PMC11675722; doi:10.3390/diagnostics14242895)
Supplement: Supplementary file 1 [file diagnostics-14-02895-s001.zip › diagnostics-3261890-supplementary.pdf]

Table S1. Clinical characteristics of the total population analyzing interactions of age and sex with glucose homeostasis and hepatic markers in prediabetics.

		<40 years			40-49 years			50-59 years			≥60 years					
Variable	Total	Women	Men	P	Women	Men	P	Women	Men	P	Women	Men	P	P sex	P age	P int.
N	2,220	282	120	-	392	133	-	415	210	-	415	253	-	-	-	-
BMI (kg/m²)	36±6	38±7	37±7	0.359	36±7	37±8	0.844	35±6	34±6	**0.015**	34±5	33±4	0.140	**0.004**	**<0.001**	0.657
WC (cm)	110±14	109±15	118±17	**<0.001**	106±14	117±23	**<0.001**	107±13	116±15	**<0.001**	106±12	115±11	**<0.001**	**<0.001**	0.151	0.318
Glucose (mg/dL)	104±13	103±11	105±12	0.106	104±11	106±14	0.162	103±12	104±13	0.251	102±12	104±16	0.340	**0.016**	0.246	0.108
Insulin (μU/L)	13±8	14±8	18±12	**0.003**	12±7	15±10	**0.002**	12±6	14±7	**<0.001**	12±6	13±6	0.053	**<0.001**	**<0.001**	**0.027**
HOMA-IR	3.4±2.1	3.8±2.4	5.0±3.6	**0.001**	3.3±1.9	4.1±3.0	**0.003**	3.1±1.9	3.9±2.1	**<0.001**	3.0±1.7	3.2±1.5	0.138	**<0.001**	**<0.001**	**0.002**
C-Pep (pmol/L)	913±360	957±385	980±424	0.581	897±367	926±374	0.440	919±365	888±367	0.305	913±341	864±305	0.062	0.320	0.060	**0.002**
HbA1c (%)	5.4±0.8	5.2±1.0	5.3±0.8	0.381	5.2±1.0	5.4±0.3	**0.017**	5.4±0.7	5.5±0.5	0.576	5.5±0.6	5.4±0.5	**0.042**	0.357	**<0.001**	0.148
TyG index	8.6±0.4	8.7±0.5	8.8±0.4	0.055	8.7±0.4	8.8±0.5	0.442	8.6±0.5	8.7±0.4	**0.044**	8.6±0.4	8.8±0.5	**0.008**	**<0.001**	0.601	0.076
TC (mg/dL)	200±39	200±37	198±34	0.715	199±39	204±41	0.249	198±38	201±39	0.476	199±37	203±41	0.160	0.113	0.135	0.677
TG (mg/dL)	133±69	131±62	137±61	0.339	132±62	138±74	0.340	133±82	139±71	0.387	125±65	137±70	**0.021**	**0.013**	0.403	0.376
HDL-c (mg/dL)	48±10	48±11	48±9	0.968	49±10	48±11	0.580	49±10	48±10	0.828	49±11	50±10	0.923	0.959	0.567	0.650
LDL-c (mg/dL)	125±32	125±231	122±28	0.434	124±34	129±35	0.114	124±32	125±33	0.683	124±31	126±34	0.430	0.276	0.287	0.792
hs-CRP (mg/L)	5.3±3.0	5.7±10.0	5.1±4.8	0.510	4.9±5.5	5.1±5.4	0.828	5.4±7.2	5.0±5.2	0.552	5.5±7.2	5.0±7.1	0.370	0.309	0.169	0.803
AST (IU/L)	27±10	24±7	32±13	**<0.001**	25±9	31±12	**<0.001**	27±9	29±12	**0.003**	28±10	29±10	0.197	**<0.001**	0.201	**<0.001**
ALT (IU/L)	28±15	22±13	41±19	**<0.001**	23±14	35±15	**<0.001**	27±14	32±17	**<0.001**	25±12	28±15	**0.013**	**<0.001**	**0.007**	**<0.001**
AST:ALT ratio	1.2±0.6	1.1±0.5	0.9±0.6	**<0.001**	1.3±0.7	1.0±0.6	**0.001**	1.2±0.5	1.0±0.5	0.058	1.3±0.8	1.2±0.6	0.148	**<0.001**	0.054	0.051
SBP (mmHg)	129±16	120±13	131±13	**<0.001**	122±15	129±12	**<0.001**	128±15	132±15	**<0.001**	135±15	136±14	0.372	**<0.001**	**<0.001**	**0.008**
DBP (mmHg)	78±11	74±11	79±11	**<0.001**	75±11	79±9	**0.001**	78±11	82±10	**<0.001**	78±10	80±9	**<0.001**	**<0.001**	**<0.001**	0.107
HSI	47±8	50±9	44±10	**<0.001**	49±9	45±9	**<0.001**	47±7	43±6	**<0.001**	47±8	43±6	**<0.001**	**<0.001**	**<0.001**	0.243
QoL score (0-100)	60±18	53±19	54±18	0.738	57±18	58±19	0.728	59±19	63±17	**0.020**	65±18	68±15	**0.009**	**<0.001**	**<0.001**	0.690
Energy intake (Kj/d)	8780±2730	9156±3603	10670±3295	**0.001**	8878±2388	10058±1077	**<0.001**	8151±2389	9616±2956	**<0.001**	7659±1920	8981±2267	**<0.001**	**<0.001**	**<0.001**	**<0.001**
CH intake (g/d)	210±74	232±96	257±85	**0.039**	219±67	240±82	**0.008**	192±68	222±76	**<0.001**	183±58	205±55	**<0.001**	**<0.001**	**<0.001**	**<0.001**
Protein intake (g/d)	92±30	90±36	112±38	**<0.001**	89±24	111±43	**<0.001**	86±24	103±32	**<0.001**	80±20	97±26	**<0.001**	**<0.001**	**0.001**	**<0.001**
Fat intake (g/d)	86±33	93±43	107±40	**0.010**	90±31	96±36	0.118	81±29	94±38	**<0.001**	74±24	85±30	**<0.001**	**<0.001**	**<0.001**	**<0.001**
Alcohol intake (g/d)	10±15	4±9	10±18	**0.001**	5±9	13±18	**<0.001**	8±14	15±19	**<0.001**	9±12	18±19	**<0.001**	**<0.001**	**0.001**	**<0.001**

Values are presented as means ± standard deviations. Bold numbers indicate P<0.05. BMI: body mass index; WC: waist circumference; SBP: systolic blood pressure; DBP: diastolic blood pressure; HOMA-IR: homeostatic model assessment of insulin resistance; C-Pep: C-peptide; TC: total cholesterol; TG: triglycerides; HDL-c: high-density lipoprotein cholesterol; LDL: low-density lipoprotein cholesterol; hs-CRP: high-sensitivity C-reactive protein; AST: aspartate aminotransferase; ALT: alanine transaminase: TyG: triglyceride glucose index; HSI: hepatic steatosis index; CH: carbohydrates
